# Skeletal improvement in patients with Gaucher disease type 1: a phase 2 trial of oral eliglustat

**DOI:** 10.1007/s00256-014-1891-9

**Published:** 2014-05-10

**Authors:** Ravi S. Kamath, Elena Lukina, Nora Watman, Marta Dragosky, Gregory M. Pastores, Elsa Avila Arreguin, Hanna Rosenbaum, Ari Zimran, Rasha Aguzzi, Ana Cristina Puga, Andrea M. Norfleet, M. Judith Peterschmitt, Daniel I. Rosenthal

**Affiliations:** 1 Massachusetts General Hospital and Harvard Medical School, Boston, MA USA; 2 Russian Academy of Medical Sciences, Moscow, Russia; 3 Hospital Ramos Mejia, Buenos Aires, Argentina; 4 Instituto Mexicano del Seguro Social Hospital de Especialidades, Col. La Raza, Mexico; 5 New York University, New York, USA; 6 Instituto Argentino de Diagnostico y Tratamiento, Buenos Aires, Argentina; 7 Rambam Medical Center, Haifa, Israel; 8 Sha’are Zedek Hebrew University and Hadassah Medical School, Jerusalem, Israel; 9 Genzyme, a Sanofi company, Cambridge, MA USA; 10 Present Address: Fairfax Radiological Consultants, Fairfax, VA USA; 11 Present Address: Yale University School of Medicine, New Haven, CT USA; 12 Present Address: Alexion Pharmaceuticals, Cambridge, MA USA; 13 Department of Radiology, Massachusetts General Hospital, 175 Cambridge Street, Boston, MA 02114 USA

**Keywords:** Gaucher disease type 1, Bone disease, Drug therapy, Targeted molecular therapy, Magnetic resonance imaging, Dual-energy X-ray absorptiometry

## Abstract

**Objective:**

Eliglustat is an investigational oral substrate reduction therapy for Gaucher disease type 1 (GD1). Its skeletal effects were evaluated by prospective monitoring of bone mineral density (BMD), fractures, marrow infiltration by Gaucher cells, focal bone lesions, and infarcts during an open-label, multi-site, single-arm phase 2 trial (NCT00358150).

**Materials and methods:**

Institutional review board approval and patient informed consent were obtained. Eliglustat (50 or 100 mg) was self-administered by mouth twice daily; 19 patients completed 4 years of treatment. All were skeletally mature (age range, 18–55 years). DXA and MRI assessments were conducted at baseline and annually thereafter. X-rays were obtained annually until month 24, and then every other year.

**Results:**

Lumbar spine BMD increased significantly (*p* = 0.02; *n* = 15) by a mean (SD) of 9.9 % (14.2 %) from baseline to year 4; corresponding T-scores increased significantly (*p* = 0.01) from a mean (SD) of −1.6 (1.1) to −0.9 (1.3). Mean femur T-score remained normal through 4 years. Femur MRI showed that 10/18 (56 %) patients had decreased Gaucher cell infiltration compared to baseline; one patient with early improvement had transient worsening at year 4. There were no lumbar spine or femoral fractures and no reported bone crises during the study. At baseline, 8/19 (42 %) patients had focal bone lesions, which remained stable, and 7/19 (37 %) patients had bone infarctions, which improved in one patient by year 2. At year 4, one new asymptomatic, indeterminate bone lesion was discovered that subsequently resolved.

**Conclusions:**

Eliglustat may be a therapeutic option for treating the skeletal manifestations of GD1.

## Introduction

Skeletal complications are a major cause of morbidity in patients with Gaucher disease type 1 (GD1) [1–3]. In GD1, a genetic deficiency of acid β-glucosidase impairs the catabolism of glucosylceramide, resulting in intracellular, particularly intralysosomal, glycolipid accumulation [4]. Enlarged, lipid-laden macrophages known as “Gaucher cells” are observed in most tissues, but heavy infiltrations occur in spleen, liver, and bone marrow, giving rise to visceromegaly, cytopenia, and diverse skeletal lesions associated with mineral loss, ischemia, and marrow packing [5, 6].

Imaging of the skeleton provides a means of evaluating GD1 severity and monitoring responsiveness to treatment. On magnetic resonance (MR) imaging, areas of reduced bone marrow signal intensity (“dark marrow”) occur in T1-weighted sequences. Progressive accumulation of Gaucher cells displaces normal adipocytes from the marrow compartment [1, 7, 8], leading to abnormal quantities and distribution of “dark marrow.” This process begins in the axial skeleton and progresses to the appendicular skeleton, advancing in the lower extremity in a predictable sequence over time [1]. Focal collections of Gaucher cells may result in lytic lesions. The mechanistic link between marrow infiltration and the development of bony complications is not clear, but extensive Gaucher cell infiltration is associated with osteopenia, bone infarctions, avascular necrosis, lytic lesions, and pathological fractures [1, 5, 9]. Chronic bone pain and acute bone crises also often accompany skeletal disease in GD1 patients.

Treatment of GD1 with enzyme replacement therapy (ERT) has been available since 1991. Enzyme replacement enhances the body’s ability to metabolize the glycolipid. It is effective, but costly, difficult to produce, and requires intravenous infusion. In 2003, miglustat (Zavesca, Actelion, Allschwil, Switzerland), a substrate reduction therapy (SRT), became available for GD1 [10–12], but due to its safety and tolerability profile, was approved only for patients for whom ERT is not an option. SRT uses daily regimens of orally administered, small-molecule inhibitors of glucosylceramide synthesis to decrease the substrate burden. ERT has been shown to improve or stabilize bone disease in many patients, particularly when treatment is initiated before irreversible pathological changes ensue [8, 13, 14]. The data for miglustat are more limited [15]. An alternative novel SRT, eliglustat, is currently in clinical development as a treatment for adults with GD1. Two-year and 4-year results of an ongoing phase 2 clinical trial have demonstrated a good safety and tolerability profile, statistically significant improvements in platelet count and hemoglobin level, decreases in spleen volume and liver volume, and increases in lumbar spine bone mineral density and decreased or stabilized bone marrow infiltration by Gaucher cells [16–18]. Here we report specifically on skeletal responsiveness to eliglustat after 4 years of eliglustat treatment in this phase 2 trial.

## Materials and methods

### Study design

The phase 2 trial was a multi-site, open-label, single-arm study sponsored by Genzyme, a Sanofi company, and registered as NCT00358150 at www.clinicaltrials.gov. The Ethics Committee or Institutional Review Board at each site approved the protocol. Patients provided written informed consent in accordance with the Declaration of Helsinki. Patient privacy was maintained in compliance with the Health Insurance Portability and Accountability Act. The main inclusion criteria were a diagnosis of GD1 confirmed by deficient acid β-glucosidase activity and Gaucher genotype, age 18 to 65 years, the presence of splenomegaly with anemia, and/or thrombocytopenia [16–18]. Patients could not have received ERT, miglustat, or corticosteroids within the previous year or bisphosphonates within the previous 3 months. The bone-related exclusion criteria were any new pathological bone involvement or bone crises in the previous year.

Of 26 patients enrolled, six discontinued before or at year 1, and one patient discontinued at year 2 [16, 17]. The reasons for patient discontinuation included asymptomatic non-sustained ventricular tachycardia (*n* = 2), pregnancy (*n* = 3), administrative withdrawal (*n* = 1), and bone lesion (*n* = 1). The patient with the bone lesion was withdrawn after the first year because of worsening osteonecrosis of the femoral head, which was asymptomatic and was detected retrospectively on screening images.

The remaining 19 patients completed 4 years of treatment. Eliglustat capsules (50 or 100 mg) were self-administered twice daily; dose selection was based on day-10 plasma drug concentrations. Spleen volume, liver volume, platelet counts, and hemoglobin values were determined as previously described [14, 15]. Patients were instructed to return any unused medication at each study visit to monitor compliance based on number of returned capsules. Only patients with compliance rates ≥80 % were included for statistical analysis in the study.

#### Bone disease assessments

**Fig. 1:**
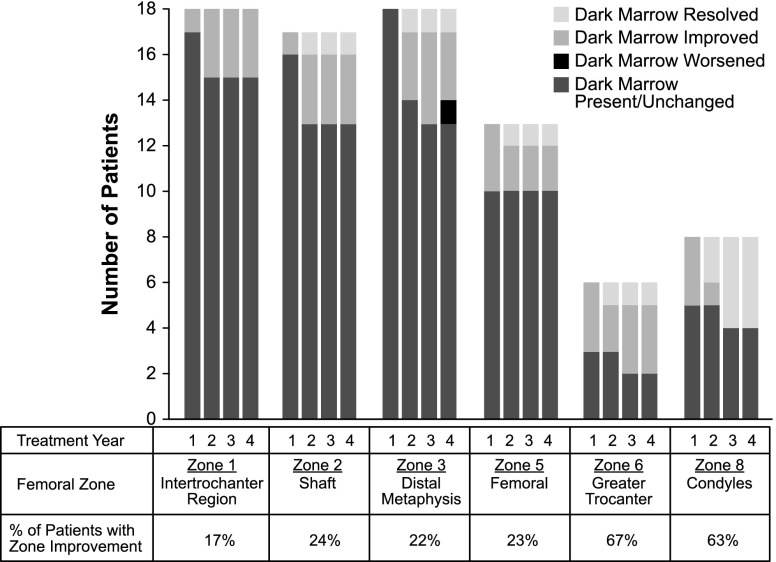
Improvements in femur dark marrow during 4 years of eliglustat treatment. The six zones of the femur are numbered according to the original method [1]

MR and dual-energy X-ray absorptiometry (DXA) images were obtained at study sites during screening and every 12 months during treatment. Plain radiography (X-ray) was done annually until year 2, and then every other year. All images were evaluated by two central reviewers who compared each result to the previous result to assess changes over time (“better,” “worse,” or “the same”) and were not blinded to patients or timepoints [19]. Images were assessed for quality and comparable anatomical sites at all time points and reviewer consensus was reported. Coronal spin-echo T1- and T2-weighted MR images of both femurs were obtained for assessment of focal marrow lesions, infarcts, and dark marrow. Marrow infiltration, or “dark marrow,” was evaluated on MR images using a modification of the Rosenthal method in which 11 anatomical zones in the lower extremity are numbered according to the sequence in which Gaucher cells infiltrate, displacing normal fatty marrow [1]. Because MR images were only obtained of the femur in this study, analysis was restricted to six zones (see Fig. 1). The presence of dark marrow on MR was visually assessed in each zone and was designated as not present, present, unchanged, decreased, or increased. DXA images of the lumbar spine and femur were obtained for measurement of bone mineral density (BMD) using Hologic or Lunar scanners. Data were excluded from analyses for four patients whose DXA examinations were not performed on equipment from the same manufacturer at all assessments. DXA results were expressed as percent change in BMD from baseline (g/cm^2^) and as T- and Z-scores [20]. Anterior-posterior radiographs of both femurs and lateral views of the thoracic and lumbar spine were reviewed for the presence of infarcts, lytic lesions, fractures, and osteonecrosis of the femoral head and condyles. Cortical thickness measurements of the mid-femoral diaphyses were made to the nearest 0.5 mm using a transparent ruler. Patient self-assessments of bone pain, bone crises, and mobility were documented at least every 6 months.

### Statistical analyses

To evaluate differences between baseline and year 4, changes or percent changes from baseline were analyzed using a paired *t* test or Wilcoxon signed-rank test, as appropriate. To evaluate associations between the change in BMD T-score and changes in other clinical parameters, Pearson’s correlation coefficients were calculated. A *p* value of 0.05 was considered statistically significant.

## Results

For the 19 study patients, the median age at initiation of treatment was 31.4 years. All patients were skeletally mature (age range, 18.6–55.7 years). The extent of splenomegaly, cytopenia, and skeletal defects at baseline was consistent with a moderate-to-severe stage of GD1 (Table 1).

**Table 1:** Demographics and baseline characteristics

Parameter	Value		*n* (%)
Gender	Female Male		10 (53) 9 (47)
Ethnicity	Caucasian, Ashkenazi Jewish Caucasian, non-Jewish Hispanic		3 (16) 14 (74) 2 (11)
Genotype	N370S/N370S N370S/L444P N370S/Other L444P/Other		2 (10) 8 (42) 8 (42) 1 (5)
Parameter*	Mean (SD)	Median	Min-Max
Age at treatment, years	33.6 (12.5)	31.4	18.6–55.7
Age at diagnosis, years (*n* = 18)	24 (13)	22.95	5–52
Number of femur zones with dark marrow (*n* = 18)	4 (2)	4.5	0–6
Lumbar spine bone mineral density, T-score (*n* = 15)	−1.6 (1.06)	−1.7	−3.1–0.6
Hemoglobin, g/dl	11.3 (1.54)	11.75	8.8–14.6
Platelet count (×1,000/μl)	68.7 (21.16)	66.5	39–105.5
Spleen volume, MN (*n* = 18)	17.3 (9.53)	13.52	10.45–49.16
Liver volume, MN (*n* = 18)	1.7 (0.42)	1.59	1.11–2.47

*n = 19 for all parameters, except where noted

*MN* multiples of normal, assuming normal liver volume is 2.5 % of body weight and normal spleen volume is 0.2 % of body weight

As expected, at least some dark marrow was present at baseline in the femurs of nearly all patients (18/19, 95 %). There was dark marrow in all six zones in five patients and in at least three zones in the other 13 patients. The intertrochanteric region, shaft, and distal metaphysis were the most commonly affected zones. During 4 years of eliglustat treatment, 10/18 (56 %) patients showed improvement in one to six zones, while the other eight (44 %) patients remained stable. At year 4, one patient exhibited worsening within one of six zones that had previously improved; however, this patient resumed improvement in dark marrow 1 year later. The patients who responded with dark marrow improvements tended to be male and to have more zones with marrow involvement, larger spleen volumes, and lower hemoglobin levels at baseline. The dark marrow responders also showed greater improvements in spleen volume, hemoglobin levels, and platelet counts at year 4 than non-responders (Table 2).

**Table 2:** Comparison of patients with and without improvement in dark marrow during 4 years of eliglustat treatment*

	Patients with improvement	Patients without improvement	*p* value
*n* (%)	10 (56 %)	8 (44 %)	
Age at baseline (years)	31 (18, 55)	31 (19, 53)	0.7557
Ratio of males to females	60:40	25:75	0.1880
Femoral zones with dark marrow			
Baseline (*n*)	5 (3, 6)	3 (3, 6)	
Change from baseline to 4 years (*n*)	−3 (−5, −1)	0 (0, 0)	0.0002
Spleen volume			
Baseline (MN)	17.0 (10.5, 49.2)	12.8 (10.8, 14.6)	
Change from baseline to 4 years (MN)	−11.2 (−30.8, −5.5)	−7.5 (−10.6, −5.6)	0.1002
Hemoglobin			
Baseline (g/dl)	10.9 (8.9, 14.6)	11.9 (8.8, 12.5)	
Change from baseline to 4 years (g/dl)	3.1 (0.4, 4.6)	1.4 (0, 2.4)	0.0329
Platelets			
Baseline (×1,000/μl)	62.5 (39.0, 105.5)	66.7 (47.0, 95.0)	
Change from baseline to 4 years (×1,000/μl)	65.5 (6.5, 212.0)	31.5 (4.0, 99.5)	0.2133

*Data are presented as median (minimum, maximum) for all parameters except the ratio of males to females. Spleen volume in multiples of normal (*MN*) is normalized to body weight, assuming normal spleen volume (ml) is 0.2 % of body weight

**Fig. 2:**
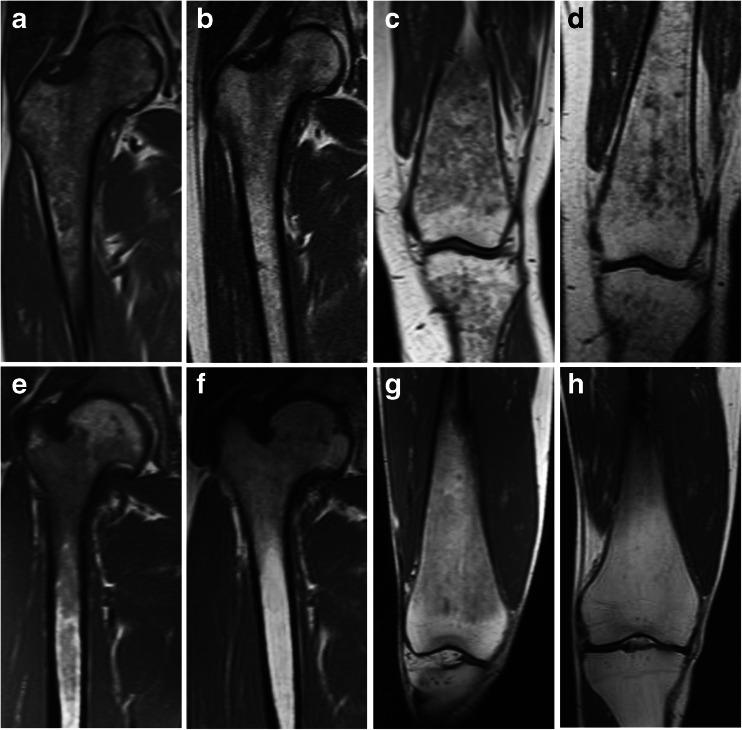
Dark marrow improvement in two patients after 4 years of eliglustat therapy. *Top* Coronal T1-weighted MR images of the proximal right femur of a 55-year-old female patient at baseline (**a**) and at 48 months (**b**) demonstrate decreased but persistent dark marrow in the femoral head epiphysis, greater trochanteric apophysis, and intertrochanteric region of the proximal femur. Dark marrow has also largely disappeared from the proximal femoral diaphysis. Coronal T1-weighted MR images of the distal left femur at baseline (**c**) and at 36 months (**d**) demonstrate substantially decreased dark marrow in the distal femoral diaphysis and metaphysis. *Bottom* Coronal T1-weighted MR images of the proximal right femur of a 30-year-old male patient at baseline (**e**) and at 48 months (**f**) demonstrate decreased dark marrow in the intertrochanteric region and proximal diaphysis of the femur. Coronal T1-weighted MR images of the distal right femur at baseline (**g**) and at 48 months (**h**) in the same patient demonstrate complete resolution of dark marrow in the distal femoral diaphysis and metaphysis with residual normal fatty marrow

Figure 1 shows that marrow improvements were apparent by the first year and continued through the fourth year of treatment. As expected, based upon previous studies, improvements in marrow involvement were most frequent in those sites tending to be affected later in the course of the disease (the greater trochanter and the condyles). Figure 2 (top) presents pre-treatment and year-4 images from a 55-year-old female with dark marrow present in all six femoral zones at baseline. After 2 years of treatment, dark marrow resolved in the greater trochanter and condyles and decreased in the other four zones. Figure 2 (bottom) presents images from a 30-year-old male with baseline involvement of four zones; after 4 years of treatment, dark marrow resolved in the shaft, distal metaphysis and femoral head, but not in the intertrochanteric region.

**Fig. 3:**
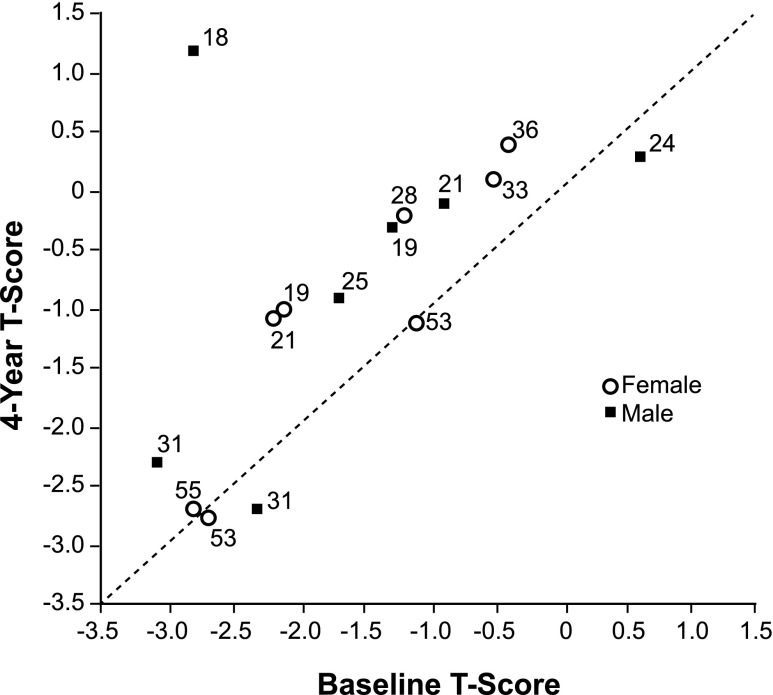
Changes in lumbar spine bone mineral density with eliglustat treatment. Each point represents the T-score for a given patient, with age at treatment initiation shown adjacent to the symbol

Lumbar spine BMD (g/cm^2^) increased significantly (*p* = 0.02) from baseline to year 4 by a mean (SD) of 9.9 % (14.2 %) in the 15 patients with data. This increase in mean (SD) BMD from 0.99 (0.15) to 1.09 (0.19) g/cm^2^ corresponded to an increase in mean (SD) T-score from −1.6 (1.1) (osteopenia) to −0.9 (1.3) (normal), and to an increase in mean (SD) Z-score from −1.17 (0.9) to −0.48 (1.1). Figure 3 displays baseline and year-4 lumbar spine T-scores for each patient and demonstrates that improvements were observed across both gender and age. Excluded from the BMD analysis were two patients assessed with different scanner types at baseline and follow-up, a patient with local bone abnormalities at screening that precluded BMD measurements, and a 40-year-old male with osteoporosis who was re-instated on bisphosphonate therapy following a decrease in lumbar spine T-score from −2.9 at baseline to −3.0 at year 1. For 13 patients with BMD data for the femur, the mean T-score increased from −0.04 (0.9) at baseline to 0.13 (1.0) at year 4; the mean Z-score increased from 0.27 (0.7) at baseline to 0.48 (0.8) at year 4. Cortical thickness of the mid-femoral diaphyses did not change from baseline in all 12 patients with data.

No existing (prevalent) fractures or new (incident) fractures were detected in the spine or femurs of any patient at baseline or during 4 years of eliglustat treatment. Thirteen focal bone lesions were observed in the femurs of 8/19 (42 %) patients at baseline. These pre-existing lesions, located in the diaphysis (*n* = 7), proximal metaphysis (*n* = 3), distal metaphysis (*n* = 2), and condyles (*n* = 1), remained stable during treatment, and no new lesions developed in any patient. Bone infarctions were present in 7/19 (37 %) patients at baseline, with a total of 12 infarctions detected in the diaphysis (*n* = 6), distal metaphysis (*n* = 2), femoral head (*n* = 2), intertrochanteric region (*n* = 1), and condyles (*n* = 1). After 2 years of treatment, two (of four) infarctions improved in a 24-year-old patient. All other pre-existing infarctions remained stable.

**Fig. 4:**
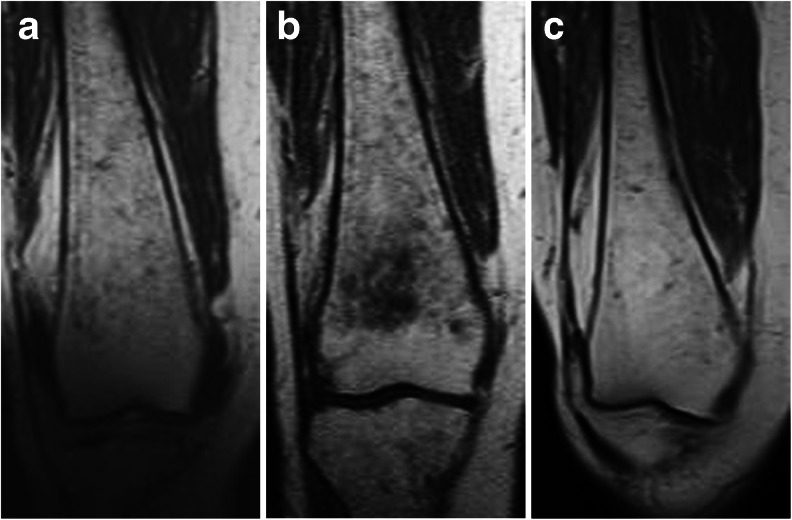
Resolution of indeterminate lesion and continued dark marrow improvement in one patient. Coronal T1-weighted MR images of the distal right femur of a 55-year-old female patient at 36 months (**a**) and 48 months (**b**) demonstrate an interval increase in dark marrow in the distal femoral diaphysis and metaphysis with a new indeterminate metaphyseal bone lesion at 48 months. At 60 months (**c**), the bone lesion resolved and dark marrow continued to improve, even as compared to 36 months, with near-complete resolution of dark marrow in the distal femoral diaphysis and metaphysis

A few instances did not fit the predominant pattern of statistically significant improvement and are of unclear significance. A new asymptomatic indeterminate bone lesion and some worsening of dark marrow were observed at year 4 in the distal femoral metaphysis of a 55-year-old female (Fig. 2, top). However, Fig. 4 shows that this lesion was not present 1 year later, and dark marrow at that location continued to improve beyond the level seen after 3 years of treatment. An asymptomatic infarction was also detected in the femoral condyle of a 49-year-old male patient, but it is not known if the lesion was present at baseline because earlier images did not include that anatomical site. During the 4-year period, patient self-assessments indicated that mobility was not restricted before or during treatment for any patient, and no patient experienced a clinically significant change in bone pain. No new bone crises were reported.

## Discussion

Treatment with eliglustat resulted in improvement in bone marrow infiltration and lumbar spine BMD that was sustained for 4 years in the majority of patients. Unlike ERT, in which enzyme is generally infused every 2 weeks to enhance catabolism of glucosylceramide, eliglustat was administered twice daily by mouth to reduce de novo synthesis of glucosylceramide. Skeletal responses induced by eliglustat were qualitatively similar to those reported for ERT with human placental and recombinant human enzyme preparations (alglucerase and imiglucerase, respectively, Genzyme, a Sanofi company) [21]. For the 4-year cohort, eliglustat treatment resulted in marrow improvements in 28 % of patients at 1 year and 56 % by the fourth year. This pattern is similar to imiglucerase, in which marrow improvements occur within the first year in a small proportion of patients and the proportion increases with prolonged treatment, although up to 30 % of patients remain unresponsive [1, 22, 23].

Marrow infiltration, or “dark marrow,” was evaluated by MRI of both femurs. With treatment, the highest percentage of dark marrow reductions occurred in zones expected to be most recently infiltrated, the condyles and greater trochanter, whereas relatively few reductions were observed in the early infiltrated intertrochanteric region. Patients with marrow improvement tended to have more extensive marrow involvement, more severe splenomegaly, and lower hemoglobin levels at baseline than patients who did not show improvement (Table 2). Infiltration of vertebral marrow has previously been associated with splenomegaly [19]. Although these may be unrelated markers of disease severity, it is possible that marrow infiltration is exacerbated by splenomegaly, as cytopenia due to hypersplenism may promote reconversion of yellow to hematopoietic marrow. Marrow responders also tended to exhibit greater improvements in spleen size and hematologic measures with treatment, although this correlation was only statistically significant for hemoglobin level, perhaps due to the small sample size (Table 2). Restoration of yellow marrow in bone could reflect the combined effect of eliglustat on Gaucher cell populations in the spleen (reducing hypersplenism) and bone marrow (restoring function to normal sites of hematopoiesis). This phase 2 study enrolled adult GD1 patients selected on the basis of an enlarged spleen (volume >10 MN) and the presence of either mild-to-moderate anemia or mild-to-severe thrombocytopenia, or both. Retrospective analyses of patients on ERT with imiglucerase have not consistently shown a correlation between marrow response and improvements in visceral and hematologic parameters, but variability in age of patients, time on treatment, spleen status, and baseline disease severity could account for these discrepancies [19, 23, 24].

Improvement in bone density occurred earlier than detectable marrow improvement. Eliglustat treatment produced substantial BMD gains in the first year, which were furthered or maintained through the fourth year. An increase in BMD was reported at 6 months for miglustat [15]. The authors of that study postulated that a small molecule may distribute to bony compartments that are less accessible to therapeutic enzymes. These observations suggest that the mineral component of the skeleton may show a more rapid response to SRT than to ERT, recognizing that the rate and magnitude of BMD responses to ERT (imiglucerase) depend on many factors, including the dose, BMD status, and age at which enzyme infusions are initiated [8, 13, 14].

Of 15 patients with data for lumbar spine BMD, 12 were osteopenic or osteoporotic at baseline, and some T-score improvement was seen in all but three patients by year 4. T-score changes did not correlate with marrow improvement (*r* = −0.29; *p* = 0.29), but did correlate with changes in platelet counts (*r* = 0.66; *p* = 0.01) and spleen volume (*r* = −0.53; *p* = 0.04). The lack of association between marrow involvement and bone density has been previously reported [7] and has suggested that cell types other than the Gaucher macrophage may play a role in GD1-associated osteopenia [25–27]. The BMD response to eliglustat could involve direct effects on osteoblasts, osteoclasts, and/or their precursors, as eliglustat is widely biodistributed [17, 28, 29]. The apparent increase in bone density seen on DXA may also reflect changes in the marrow composition. Additional data will be required to see if the less reactive cortical bone also demonstrates increasing density over time. The correlation of BMD improvement with platelet recovery or spleen volume reduction should not be assumed to imply causality. Eliglustat produced substantial improvements in all measures of disease in several patients. The largest BMD increase after 4 years of treatment was seen in an 18-year-old male patient whose lumbar spine T-score rose from osteoporotic to normal range (from −2.8 to 1.2) with normalization of the Z-score (−2.2 to 1.0), accompanied by marrow improvements in three of six involved zones and recoveries in platelets (from 70 to 282 × 1,000/μl), hemoglobin (from 11.9 to 15.1 g/dl), and spleen size (from 28.5 to 2.5 multiples of normal [MN]). It is possible that increases in BMD may be due to generally improved health status and activity level. A few patients with abnormal baseline BMD did not gain skeletal mass, but did exhibit other treatment benefits. A 31-year-old male patient whose lumbar spine T- and Z-scores both decreased from −2.3 to−2.7 had improvements in platelets (103 to 161 × 1,000/μl), hemoglobin (12.8 to 14.8 g/dl), spleen size (10.5 to 4.2 MN), and dark marrow (see Fig. 2 bottom). It is not clear why some patients fail to gain bone mass, but many factors other than those associated with GD1 may influence bone formation and could mask or limit the effect of treatment. Regardless of etiology, low BMD is recognized as a strong risk factor for fractures of the spine and femur in GD1 [30]. There were no new fractures among our patients.

It has been shown that ERT reduces the incidence of bone crises and bone pain associated with skeletal lesions [13, 31]. The patients who completed 4 years of treatment did not report any bone crises, and no symptomatic infarctions were identified on imaging. Two pre-existing infarcts improved in one patient. Although this result is encouraging, conclusions cannot be drawn about a treatment effect since spontaneous healing of osteonecrosis has been reported [32].

We do not have an explanation for the asymptomatic indeterminate lesion that appeared in the distal femoral metaphysis in one patient. It was accompanied by re-appearance of dark marrow in the same location (although not in the other five zones) (Fig. 2, top). The lesion was not present on images obtained 1 year later, the dark marrow in this region had resumed its improvement, and all hematologic and visceral parameters showed continuous improvement (Fig. 4).

The main limitations of this trial are the small number of patients, especially given the clinical heterogeneity of GD1; the uncontrolled study design of this proof-of-concept phase 2 trial; and the unblinded reviews of the bone images. However, this trial provides useful and objective information about the long-term effect of eliglustat on bone manifestations of Gaucher disease. Additional data from an ongoing phase 3 randomized, controlled trial in treatment-naive adults with GD1 (ENGAGE) will provide further insight into the effect of eliglustat on hematologic, visceral, and bone parameters.

## Conclusions

The results of radiologic assessments of bone suggest that eliglustat, an investigational oral SRT, may be a therapeutic option for treating the skeletal manifestations of GD1. An initial comprehensive radiologic evaluation of GD1 patients for marrow and osseous complications should form the basis for monitoring the effects of eliglustat treatment at regular intervals.
